# Distinct Patient Characteristics and Risk Profiles in Upper vs. Lower Leg Fractures: Insights from a Comprehensive Cohort Study

**DOI:** 10.3390/diagnostics16010011

**Published:** 2025-12-19

**Authors:** Felix Erne, Christoph Ihle, Sabrina Ehnert, Tina Histing, Andreas K. Nüssler, Elke Maurer

**Affiliations:** 1 Siegfried Weller Research Institute, BG Unfallklinik, Department of Trauma and Reconstructive Surgery, Eberhard Karls University Tuebingen, Schnarrenbergstr. 95, 72076 Tuebingen, Germany; 2 Department of Orthopedics and Traumatology, Diakonie-Klinikum Hospital, Rosenbergstr. 38, 70176 Stuttgart, Germany; 3 M&I-Fachkliniken Hohenurach, Orthopädische Klinik, Immanuel-Kant-Str. 33, 72574 Bad Urach, Germany; 4 Lumedis Orthopedics, Kirchstr. 14, 60311 Frankfurt, Germany

**Keywords:** lower extremity fractures, postoperative complication, malnutrition, Clavien–Dindo, injury severity, risk factor, complication rates, trauma surgery, orthopedics, risk stratification

## Abstract

**Background and Purpose**: Surgical treatment of lower-extremity fractures can result in postoperative complications. Irrespective of specific surgical procedures, postoperative outcomes may be influenced by pre-existing comorbidities, as well as by the severity of the injury. This study investigates whether (I) internal factors and (II) external factors are associated with (III) postoperative complication rates. **Material and Methods**: A prospective study was conducted at a Level I trauma center between 2014 and 2018. A cohort of 416 patients with surgical treatment after traumatic long bone fractures of the lower extremities was assessed, with comorbidities along with factors such as age, malnutrition, and impaired mobility systematically evaluated. Injury severity was classified using the Abbreviated Injury Scale; fracture type and location using the AO/OTA classification; and postoperative complications using the Clavien–Dindo system. **Results**: Across the cohort, internal factors such as immobility and malnutrition were associated with an increased complication rate. Age showed a weak correlation. Obesity demonstrated a trend toward a protective effect. Patients with femoral fractures exhibited a higher prevalence of cardiovascular comorbidities, with heart failure representing the most pronounced risk factor. In the subgroup with lower leg fractures, chronic heart disease also remained a significant predictor of complications. Correlation analysis further revealed a significant positive association between injury severity and the occurrence of complications across the overall cohort. **Conclusions**: This study provides valuable insights into risk factors for complicated postoperative courses. Injury severity appears to be a promising predictor of complication risk in patients with leg fractures. Reduced mobility and malnutrition, likewise, were significantly associated with increased complication rates; these may represent the most readily addressable modifiable risk factors. All data were collected using validated, user-friendly classification systems that may be suitable for predictive modeling.

## 1. Introduction

Injuries to the lower extremities are often associated with severe limitations for the affected person, like profound functional impairment and substantial decline in quality of life. Fractures of the leg represent a substantial portion of orthopedic trauma cases, contributing significantly to both morbidity and healthcare burden worldwide [1,2]. Depending on the location of the fracture in the leg, different specific risks and particular complications have been described [3,4,5]. From a trauma-surgical perspective, classical disease models often place primary emphasis on the mechanical aspects of the traumatic event and the extent of the resulting injury. This focus is appropriate, as it represents the most urgent, clearly visible, and treatment-relevant problem. It forms the basis for determining the necessity of surgical intervention, as well as guiding the practical approach to operative management. But recovery after orthopedic surgery is influenced by various factors.

Extrinsic patient-related factors encompass external influences, including the location of fractures and the overall severity of injuries. Increasing fracture complexity has been shown to create unfavorable biomechanical environments at the fracture site, underscoring the clinical relevance of understanding fracture morphology in treatment planning [6]. Intrinsic patient-related factors are characteristics inherent to the individual, such as sex, age, and comorbidities. At the time of injury, these determinants are already irreversibly established. To systematically assess complication risk, it is essential to translate comprehensive medical histories into structured and analyzable data. Standardized anamnesis and the use of validated classification systems enable objective documentation of patient-related characteristics.

Comorbidities that may adversely affect inflammatory responses and tissue regeneration following trauma and surgery could be of relevance in lower-extremity injuries due to their impact on tissue repair. Cardiovascular comorbidities create conditions that may impair systemic and peripheral perfusion, alter hemodynamic stability, and increase the risk of perioperative complications. Peripheral arterial disease and chronic venous insufficiency are particularly relevant in lower-limb trauma, as they are directly associated with compromised arterial inflow, venous return, and impaired wound and fracture healing [7,8,9]. Pulmonary diseases elevate the risk of postoperative pulmonary complications. Hypoxemia and chronic inflammation may further delay tissue repair and increase susceptibility to infectious complications [10]. Gastrointestinal and hepatobiliary disorders are associated with malabsorption, altered protein metabolism, and systemic inflammatory states. These pathophysiological mechanisms may compromise nutritional reserves, immune response, and collagen synthesis, ultimately impairing fracture consolidation and soft tissue healing [11,12,13]. Renal disorders are linked to electrolyte disturbances, anemia, impaired bone metabolism, and immune dysfunction, all of which may negatively influence surgical outcomes and increase the risk of postoperative complications [14,15,16].

Most of the key factors named above cannot be reversed at the time of injury. A subset of risk factors, however, is considered modifiable and may, therefore, be amenable to targeted intervention. These risk factors include, for example, nutritional status and mobility status. In orthopedic surgery, malnutrition has been linked to higher complication rates in elective procedures, and emerging data in trauma populations show similar effects [17,18]. A prospective cohort study reported that 12% of severely injured patients were malnourished at ICU admission and that 70% developed malnutrition during hospitalization, with significantly higher complication rates in affected patients [19]. Multimodal interventions targeting nutrition and muscle preservation have been shown to improve clinical outcomes [20]. Functional mobility represents another critical, potentially modifiable factor. Lower-extremity trauma leads to marked reductions in physical independence, and pre-existing impairments may amplify the risk of postoperative complications. A multicentered cross-sectional study of 5511 patients demonstrated that independent ambulation was strongly associated with discharge home, while reduced mobility correlated with the need for nutritional interventions and inadequate oral intake [21].

All of the factors mentioned above are important determinants in their own right and should, therefore, be incorporated into any potential risk adjustment. This study adopts a generalized analytical perspective on injuries of the upper and lower leg. We aim to investigate whether (I) intrinsic patient-related factors and (II) extrinsic injury-related factors are associated with (III) postoperative complication rates. We hypothesize that both non-modifiable patient characteristics present at hospital admission and modifiable risk factors, particularly increased risk of malnutrition and reduced mobility, are significantly associated with higher postoperative complication rates. The results of this study may contribute to improved risk stratification, individualized perioperative management, and optimized patient outcomes.

## 2. Materials and Methods

### 2.1. Setting and Study Design

A prospective study with a standardized data collection protocol was introduced at a Level I trauma center in southern Germany in 2014. The study protocol included a diverse range of patients, reflecting the broad spectrum of trauma cases. Patients who provided informed consent were thoroughly assessed for risk factors potentially linked to postoperative complications. In a second step, analysis excluded patients who were treated conservatively without the need for surgery, as well as those who underwent elective surgical procedures for non-traumatic conditions, such as joint replacement surgeries. All postoperative interventions within our institution are guided by rigorously standardized follow-up protocols designed to ensure uniformity of care and alignment with established evidence-based practices. Consequently, individuals who had previous outside surgery and required additional reoperation at our university hospital were excluded from the study in an additional step. The subsequent analysis included all patients sustaining fractures of the femur or crural bones, whereas individuals with fractures at any other anatomical location were excluded to ensure cohort homogeneity.

### 2.2. Classification of Internal Patient-Related Risk Factors

A comprehensive data collection was carried out by screening the available treatment records and a structured patient survey by doctors and medical assistants. The following parameters were selected to better characterize each patient: age; sex; height and body weight, including body mass index; smoking status; mobility; comorbidities, including Charlson Comorbidity Index (CCI); and nutritional status using the Nutritional Risk Screening 2002 (NRS). For data collection, one-on-one interviews were offered, and standardized data acquisition was implemented through structured questionnaires. To ensure dataset integrity and minimize potential screening gaps, any missing clinical findings were proactively traced and formally requested.

### 2.3. Classification of External Patient-Related Risk Factors

To classify fractures, the AO/OTA system was employed, allowing for a standardized approach to identifying the location and type of fractures. The classification was established by the AO Foundation and the Orthopedic Trauma Association. The clinical use of the AO/OTA system also derives accurate diagnosis and treatment strategies [22]. This methodology enables a scientifically rigorous and objective assessment of parameters relevant to fracture characterization.

The Abbreviated Injury Scale (AIS) was used to classify the overall severity of injuries based on their potential risk of lethality. It provides an objective assessment of patients with multiple injuries. Briefly, injuries are categorized into the following six severity levels: AIS 1 represents minor injuries, while AIS 6 indicates fatal injuries. This classification is independent of the type, quality, or duration of treatment, aiming to group injuries with similar lethality and objectively evaluate survival probabilities [23].

### 2.4. Classification of Postoperative Complications

Postoperative complications were categorized using the Clavien–Dindo Classification (CDC), a widely accepted five-grade system. It is very well suited for comparing postoperative complications of different types of operations without describing the respective operations in detail, as surgical success is primarily assessed based on the planned post-treatment regime. CDC ranges from minor deviations requiring no significant intervention (Grade I) to life-threatening conditions (Grade IV) or death (Grade V). Grade I describes everyday clinical routine treatments like additional painkillers, antiemetics, or physiotherapy [24,25,26]. Therefore, patients classified as Grade 1 were counted as non-complicative.

### 2.5. Data Analysis and Statistics

All collected data were pseudonymized to ensure confidentiality. The dataset was imported into SPSS (IBM SPSS Statistics 29) for further analysis. The Shapiro–Wilk test was applied to assess the normality of the data distribution. For normally distributed data, statistical comparisons were performed using the independent *t*-test, while for data that did not follow a normal distribution, the Mann–Whitney U test was used for comparisons. Statistical significance was considered when the *p*-value was less than 0.05, with a predetermined alpha level of 0.05 for hypothesis testing.

Demographic and descriptive data were presented as percentages or means with standard deviations. The severity of the injury and complication rates were categorized ordinally and expressed as percentages (proportions).

To examine the relationship between nominal variables, the Chi-square test was applied to assess whether a significant association exists between the categories of the two variables. For binary nominal variables, the Odds Ratio was calculated to quantify the odds of an event occurring in one group compared to another. The *p*-value was calculated using the Chi-square test, with Fisher’s Exact Test applied when the expected frequency in any cell was less than 5.

Ordinal variables were analyzed using Spearman’s rank correlation analysis. For continuous variables, bivariate correlation analysis was performed to assess the strength and direction of the relationship. The strength of the correlation was interpreted according to Cohen [27], where |r| ≥ 0.10 represents a weak correlation, |r| ≥ 0.30 indicates a moderate correlation, and |r| ≥ 0.50 denotes a strong correlation.

## 3. Results

### 3.1. Descriptive Characteristics of the Study Population

**Figure 1:**
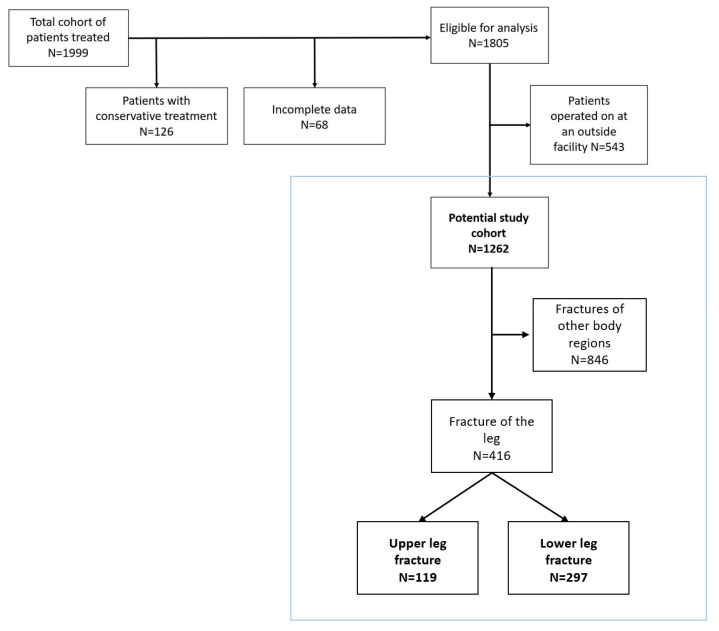
Patient’s flow chart.

The structured database included records for 1999 patients (2014 to 2018) with fractures and inpatient treatment. In total, 194 participants were excluded from the statistical analysis, with 68 removed due to missing information and 126 ruled out because conservative treatments were outside the scope of the study. As a result, 1805 patient records were deemed eligible for further analysis. A total of 543 study participants had undergone prior surgical treatment in other hospitals according to external standards and were excluded. Of the remaining 1262 patients, 416 patients had fractures of the lower extremity and were included in the study. Of these, 119 had fractures of the upper leg and 297 had fractures of the lower leg (Figure 1).

An analysis of demographic parameters revealed that patients with femur fractures were significantly older than those with lower leg fractures. They showed an average age difference of approximately 15 years (femur: 65.3 ± 19.2 years vs. lower leg: 50.1 ± 16.0 years). While the proportion of femur fractures was evenly distributed between sexes, lower leg fractures were more common in men, accounting for approximately 60% of all cases. Smoking prevalence, defined as patients with a history of exposure to smoking and measured in packyears (PY), was higher among patients with lower leg fractures compared to those with femur fractures (femur: 42.9%, lower leg: 51.5%) [28].

In terms of the burden of comorbid conditions, nearly 80% of patients with lower leg fractures scored zero on the CCI, indicating they had fewer comorbidities. However, over 50% of patients with femur fractures had a CCI score of 1 or higher (score 1–2: 36.1%, score 3–4: 10.9%, score > 4: 5.0%). Furthermore, 36.1% of patients with femur fractures were identified as being at risk for malnutrition, compared to only 13.8% of those with lower leg fractures. Detailed demographic characteristics, along with descriptive data on mobility, nutritional status, and smoking habits, are presented in Table 1.

The severity of injuries was assessed using the AIS. Within our cohort, only injuries classified as AIS 1 to AIS 3 were observed. Most patients with lower leg fractures sustained moderate injuries (AIS 2), whereas femur fractures were more frequently associated with severe injuries (AIS 3) (Table 2). A notable finding is the substantial difference in complication rates between patients with femur fractures and those with tibia fractures, reported at 42.9% (51 patients) and 24.9% (74 patients), respectively (Table 2).

According to the Clavien–Dindo classification, 202 patients were assigned to Category 0, while 89 were placed in Category I. Furthermore, 57 patients were classified as Category II, 43 as Category III, and 25 as Category IV, with no patient falling into Category V (Table 2). For the purposes of this study, patients in categories 0 and I were combined, as standard postoperative measures such as antiemetics, antipyretics, analgesics, electrolyte therapy, and physiotherapy were not regarded as complications within this cohort.

### 3.2. Correlation of Injury Severity and Patient Characteristics

In patients with femur fractures, rheumatoid arthritis (r = 0.22, *p* = 0.014) exhibited a moderate positive correlation with fracture severity, indicating that patients with rheumatoid arthritis are more likely to experience more severe femur fractures. Obesity showed a moderate negative correlation with fracture severity (r = −0.28, *p* = 0.002), suggesting that obese patients with femur fractures may experience less severe injuries, a finding that warrants further investigation. Hypertension was weakly correlated with fracture severity (r = 0.19, *p* = 0.040), indicating a modest association between hypertension and more severe femur fractures. Lastly, diabetes had a weak, non-significant correlation with fracture severity (r = 0.10, *p* = 0.30), implying that diabetes does not significantly influence the severity of femur fractures (Table 3).

In patients with lower leg fractures, several comorbidities showed significant correlations with fracture severity. Chronic obstructive pulmonary disease, chronic inflammatory bowel disease, and renal insufficiency were weakly negatively correlated with fracture severity, suggesting that patients with these conditions may experience less severe lower leg fractures. Diabetes showed a stronger negative correlation with fracture severity (r = −0.25, *p* < 0.001), suggesting that patients with diabetes tend to have more severe lower leg fractures (Table 3).

A correlation analysis (Table 4) was performed to assess the relationship between patient characteristics and injury severity in the total cohort and, furthermore, in patients with leg fractures. The results showed a significant positive correlation between age and injury severity (r = 0.35, *p* < 0.001) in patients with femur fractures, indicating that older age is associated with more severe injuries. A moderate positive correlation was also found between male sex and injury severity (r = 0.25, *p* = 0.007), suggesting that male patients tend to experience more severe femur fractures. Conversely, both size and BMI were negatively correlated with injury severity, with r-values of −0.31 (*p* < 0.001) and −0.30 (*p* < 0.001), respectively, implying that larger patients and those with a higher BMI had less severe femur injuries on average.

Smoking showed no significant correlation with injury severity (r = −0.02, *p* = 0.85), suggesting that smoking does not appear to influence the severity of femur fractures. Immobility also showed a weak, non-significant correlation (r = 0.15, *p* = 0.10), indicating that immobility may have a minor impact on injury severity, but the association was not strong enough to reach statistical significance. However, comorbidities, as measured by the CCI (r = 0.24, *p* = 0.008), demonstrated a moderate, significant positive correlation with injury severity, suggesting that more multi-morbid patients tend to experience more severe femur fractures. Malnutrition showed a weak, non-significant correlation with injury severity (r = 0.17, *p* = 0.07), indicating a potential association that did not reach statistical significance in this analysis.

In contrast, the correlation analysis in patients with lower leg fractures revealed different results. Smoking was weakly correlated with injury severity but not significantly (r = −0.07, *p* = 0.25), suggesting that smoking has little to no effect on the severity of lower leg fractures. Immobility, however, showed a significant negative correlation with injury severity (r = −0.14, *p* = 0.017), indicating that patients with greater immobility may experience less severe lower leg fractures, though the strength of the association was relatively weak. The burden of multi-morbidity, as measured by the CCI, had a strong and statistically significant negative correlation with injury severity (r = −0.22, *p* < 0.001). Finally, malnutrition showed a weak, non-significant correlation with injury severity (r = 0.09, *p* = 0.14), indicating no meaningful association in this cohort (Table 4).

### 3.3. Correlation of Complication Rates and Severity of Injury

The AIS classification provides a standardized framework for the comparable and quantitative assessment of injury severity. The correlation analysis between injury severity and the occurrence of complications in leg fractures revealed a significant positive association in the overall cohort (r = 0.26; *p* < 0.001). This indicates that greater injury severity is associated with an increased likelihood of complications. More detailed subgroup analysis revealed a slight imbalance in the correlation between injury severity and postoperative complication rate. In the subgroup of femur fractures, a similar but slightly stronger correlation was observed (r = 0.28; *p* = 0.002), suggesting a robust association within this fracture group. A significant, though somewhat weaker, positive correlation was also found in the subgroup of tibia fractures (r = 0.17; *p* = 0.003).

### 3.4. Correlation of Complication Rate and Patient Characteristics

The correlation analysis between age and complication rate in the total cohort revealed a significant but weak to moderate correlation (r = 0.15, *p* < 0.001), suggesting that age was weakly associated with an increased risk of complications across all patients. In patients with femoral fractures, a moderate positive correlation was observed between age and complication rate (r = 0.20, *p* = 0.03), indicating that older age was moderately linked to a higher complication rate in this group. However, in patients with lower leg fractures, the correlation between age and complications was weak and not significant (r = 0.037, *p* = 0.53), implying that age may not play a substantial role in predicting complications for this type of fracture.

In the overall cohort, neither sex, height, nor BMI demonstrated a statistically significant association with complication rates. Specifically, gender was not associated with increased odds of complications in the total population (OR 1.3; 95% CI 0.8–1.9; *p* = 0.34), nor in subgroups with femur (OR 1.8; 95% CI 0.9–3.7; *p* = 0.11) or lower leg fractures (OR 0.9; 95% CI 0.5–1.5; *p* = 0.65).

In contrast, immobility emerged as a significant predictor of postoperative complications across the cohort (OR 2.7; 95% CI 1.7–4.4; *p* < 0.001), with notable variation between fracture types. The strongest association was observed in patients with femur fractures (OR 2.9; 95% CI 1.3–6.2; *p* = 0.007), underscoring the critical impact of impaired mobility in this group. A significant, albeit weaker, association was also present in lower leg fracture patients (OR 2.0; 95% CI 1.0–3.9; *p* = 0.043).

Malnutrition was independently associated with increased complication risk in both femur (OR 2.3; 95% CI 1.1–4.9; *p* = 0.033) and lower leg fractures (OR 2.2; 95% CI 1.1–4.4; *p* = 0.027), as well as in the overall cohort (OR 2.6; 95% CI 1.6–4.3; *p* < 0.001). These findings highlight the importance of nutritional status as a consistent and robust predictor of adverse outcomes, regardless of fracture location (Table 5).

Comorbidity burden, as assessed by the Charlson Comorbidity Index (CCI), showed a significant association with complications in the total cohort (OR 1.6; 95% CI 1.2–2.1; *p* < 0.001) and in patients with femur fractures (OR 1.8; 95% CI 1.1–2.8; *p* = 0.013). However, this association was not statistically significant in the lower leg fracture group (OR 1.2; 95% CI 0.8–1.8; *p* = 0.29), suggesting that comorbidity burden plays a more prominent role in predicting complications among femur fracture patients. The comorbidities were subsequently examined in detail. Femur fracture patients have a higher prevalence of hypertension, coronary heart disease, heart failure, heart valve disease and cardiac arrhythmia, suggesting a greater burden of cardiovascular comorbidities in this group (Table 6).

In the unstratified analysis, several comorbidities emerged as significant predictors of increased complication risk. The most pronounced risk was associated with heart failure (OR 7.2; 95% CI 2.5–20.5; *p* < 0.001), followed by chronic heart disease (OR 3.9; 1.8–8.4; *p* < 0.001), renal insufficiency (OR 4.6; 1.7–12.7; *p* = 0.003), cardiac arrhythmia (OR 2.3; 1.1–5.0; *p* = 0.030), and diabetes mellitus (OR 2.1; 1.1–4.0; *p* = 0.026). Interestingly, obesity was associated with a significantly reduced risk (OR 0.3; 0.1–0.7; *p* = 0.005), possibly reflecting a protective “obesity paradox.”

Patients with femoral fractures demonstrated a high complication risk associated with heart failure (OR 6.1; 1.2–30.3; *p* = 0.026) and renal insufficiency (OR 4.6; 1.1–18.2; *p* = 0.027). Although chronic heart disease (OR 2.6; 1.0–6.8; *p* = 0.06) and diabetes mellitus (OR 2.1; 0.8–6.5; *p* = 0.15) approached statistical significance, they did not reach conventional thresholds. Notably, obesity also reached statistical significance, with a protective effect (*p* < 0.001), though no OR was provided.

Among patients with lower leg fractures, heart failure (OR 6.5; 1.6–26.6; *p* = 0.010) and chronic heart disease (OR 4.0; 1.0–15.2; *p* = 0.044) remained significant predictors of complications. Other comorbidities, including renal insufficiency (OR 2.0; *p* = 0.44) and diabetes mellitus (OR 1.7; *p* = 0.20), showed nonsignificant associations. Obesity again demonstrated a trend toward a protective effect (OR 0.6; *p* = 0.20), though this was not statistically significant (Table 7).

## 4. Discussion

### 4.1. Descriptive Characteristics of the Study Population

Many of the established findings from the existing literature are supported by this study, including the markedly altered patient demographics with respect to age and comorbidities among individuals with upper- and lower-leg fractures. In older individuals, traumatic femoral fractures predominantly involve the proximal femur and are most commonly the result of low-energy mechanisms. By contrast, younger adults exhibit a higher incidence of diaphyseal fractures, injuries sustained outdoors, and a greater frequency of associated traumas [2,29,30,31]. Larsen et al. describe that tibial shaft fractures are most frequently observed in males, particularly between the ages of 10 and 20, whereas in females, they occur most commonly between the ages of 30 and 40. Most injuries occur during walking, indoor activities, or sports [32]. The overall younger patient population with lower leg injuries also accounts for the lower prevalence of comorbidities, as many of these conditions increase with age.

### 4.2. Correlation of Injury Severity and Patient Characteristics

Age significantly correlated with increased severity in femur fractures. The findings are consistent with the literature, which shows age to be a significant risk factor for both the incidence and severity of femur fractures [33]. Furthermore, male sex also showed a moderate positive correlation, which aligns with findings that men tend to experience more severe fractures, likely due to higher trauma exposure [34]. Interestingly, taller patients and those with a higher BMI experienced less severe femur fractures, suggesting that increased body mass may offer some protective effect constellation. However, smoking and immobility did not show significant correlations with injury severity. Comorbidity burden, as assessed by the CCI, was positively correlated with injury severity, which concurs with the understanding that patients with more comorbidities tend to experience worse outcomes following severe fractures [35,36,37]. Injury severity was found to be higher in patients with femur fractures compared to those with lower leg fractures. For lower leg fractures, immobility showed a weak but significant negative correlation with injury severity. Smoking and malnutrition had little effect on injury severity, which could reflect the typically lower-energy trauma mechanisms and less complex healing processes in these injuries. In the future, the individual risk factors, in combination with the fracture type, could be used to better adapt surgical treatment strategies. Further studies are needed to determine which measures are most successful. Our findings also highlight the known risk of complications from malnutrition [38]. This is particularly noteworthy, as malnutrition—unlike many other risk factors—is readily identifiable through simple screening tools and, more importantly, amenable to prompt and effective intervention.

Comorbidity burden was significantly correlated with injury severity in patients with femur fractures, suggesting that multi-morbid patients are more likely to experience severe fractures. However, further clarification is needed to determine whether the risk factors themselves influence outcomes through various metabolic pathways or if impaired physical function and increased fall rates are the underlying connecting factors [39,40,41]. In contrast, the CCI showed a strong negative correlation with injury severity in patients with lower leg fractures. This observation suggests that patients with a higher comorbidity burden tend to experience less severe fractures of the lower leg. One possible explanation for this inverse relationship could be the higher incidence of high-energy trauma in this group, which may result in more severe fractures in otherwise healthier individuals. Comorbidities may not always affect fracture severity. The injury mechanism could be a more crucial factor than the patient’s overall health. Thus, previous studies have suggested that high-energy trauma is more likely to cause severe fractures regardless of the comorbidity profile of the patient [42,43,44]. Furthermore, in our cohort, patients with femur fractures tend to have a higher prevalence of cardiovascular comorbidities such as hypertension, coronary heart disease, heart failure, heart valve disease, and cardiac arrhythmia, most likely due to their higher age. These observations could be the starting point for further distinguishing between patients with upper versus lower leg fractures. Contrarily, a study identified different risk factors than ours and found that these factors were associated with higher fracture risk and mortality primarily in males, but not in females. Specifically, in males, a higher number of comorbidities, including diabetes, kidney disease, cancer, and neurological conditions, was linked to an increased risk of subsequent fractures and mortality [45]. In this context, our findings are partially consistent, as heart failure and renal insufficiency were associated with an increased risk of complications in patients with femur fractures. However, no such correlation was observed in patients with lower leg fractures.

### 4.3. Correlation of Complication Rates and Severity of Injury

The correlation analysis between injury severity and the occurrence of postoperative complications in leg fractures revealed a significant positive association. Injury severity appears to be a promising predictor of complication risk in patients with leg fractures. These findings are consistent with results from other scientific studies, such as those by Becker et al. The authors reported that the development of complications was significantly influenced by severe extremity injuries. However, they also noted that the length of stay in the intensive care unit was associated with the complication rate [46]. In contrast to Becker et al., our analysis examines a cohort of patients treated outside the intensive care unit, namely on the general ward. In our cohort, most patients with lower leg fractures sustained moderate injuries, whereas femur fractures were more frequently associated with severe injuries. Only injuries classified as AIS 1 to AIS 3 were observed, though this represents only a snapshot of actual hospital reality. Another noteworthy finding is the substantial difference in complication rates between patients with femur fractures and those with tibia fractures. This finding may likewise be indicative of the cohort’s older and more medically compromised patient profile and should, thus, be interpreted with appropriate caution.

### 4.4. Correlation of Complication Rate and Patient Characteristics

Our patient cohort with femur fractures was significantly older than those with lower leg fractures, with a mean age difference of approximately 15 years. In our cohort, age was significantly correlated with complication rates in patients with femur fractures, whereas no such correlation was found in those with lower leg fractures. This finding is consistent with previous research, which has shown that older age is a significant risk factor for complications and associated with worse functional outcomes in patients with femur fractures [2,47,48]. Lower leg fractures in younger populations are more commonly attributed to high-energy trauma, such as sports injuries or accidents. These lifestyle-attributed injury mechanisms may not carry the same risk of complications as fractures in older adults [49,50,51]. Furthermore, patients with lower leg fractures face their own unique set of challenges, such as soft tissue injury, compartment syndrome, and a higher rate of malunion or nonunion [49,51,52,53,54,55]. There was an equal distribution between sexes regarding femur fractures, while lower leg fractures were more prevalent in men. This finding aligns with the existing literature, which reports that hip and femur fractures predominantly occur among women of older populations [48,56,57]. The equal representation of both sexes in our cohort was likely due to the lack of differentiation between proximal, shaft, and distal fractures. To keep the significance of the statistical analysis as high as possible, a further subgroup analysis would no longer have been expedient. According to the literature, femoral fractures account for approximately 10% (proximal: 11.6%, shaft: <1%, distal: 3–6%) of all adult fractures, with the incidence markedly rising among older adults [58,59,60,61,62]. The incidence of femoral fractures increases with age, particularly in individuals over 65, due to factors such as osteoporosis and a higher likelihood of falls [2,47].

### 4.5. Prospective Strategies for Risk-Adjusted Interventions

Modifiable risk factors are often focused on, as interventions targeting these factors could affect patient outcomes. Within the broader context of the findings, three clinically relevant and modifiable risk factors—smoking, malnutrition, and mobility—warrant focused consideration, as they are amenable to targeted intervention.

Smoking is known to have detrimental effects on bone metabolism and regeneration, which can significantly impair bone healing. Cigarette smoke promotes osteoclast activity, leading to osteoporosis and impaired fracture healing [63,64]. Additionally, smoking contributes to systemic inflammation, which adversely impacts the cytokine profile and immune response [65]. The negative effects of smoking on fracture healing are linked to decreased osteogenesis, reduced angiogenesis, and delayed vascularization [66]. Although smoking is a well-established risk factor for impaired bone healing and delayed fracture recovery, the correlation with complications in patients with lower leg fracture was weak and absent in patients with fractures of the femur. Body regions with terminal blood supply, such as the foot and toes, were excluded from the statistical analysis. It is possible that the inclusion of these body regions would have shown a stronger effect of smoking in the statistics.

Reduced mobility is a common characteristic in geriatric patients and might lead to a vicious cycle, where diminished physical activity accelerates the onset of osteoporotic changes and sarcopenia [67,68,69,70]. Both impair the ability to recover, increase bone fragility, and predispose individuals to fractures even with minor trauma [71,72]. Inactivity has been identified as a significant risk factor for delayed fracture healing [73,74]. Previous studies have shown that impaired mobility can lead to an increased risk of complications such as deep vein thrombosis, pneumonia, and delayed wound healing [48,75,76]. There is an association with increased frailty, which, in turn, elevates the likelihood of adverse outcomes [77,78]. In our study, reduced mobility was significantly associated with an increased risk of complications. The strongest correlation was observed in patients with femur fractures. This finding aligns with the existing literature suggesting that mobility impairment is a critical factor influencing postoperative outcomes and complication rates [79].

Malnutrition is a known risk factor for poor fracture healing and increased complication rates, particularly in the elderly population [80,81,82]. In our cohort, 36.1% of patients with femur fractures were identified as being at risk for malnutrition, compared to only 13.8% of those with lower leg fractures. Malnutrition was significantly associated with an increase in complications. The rates observed in this study may similarly be attributable to the specific characteristics of the patient population and align with findings reported in the current literature. Our findings underscore the importance of nutritional assessment and intervention in patients with femur fractures to optimize recovery outcomes. Malnutrition may represent the most readily addressable modifiable risk factor and should, therefore, be prioritized for early identification and intervention.

### 4.6. Clinical Implications and Potential Intervention Options

The identification of modifiable risk factors highlights several opportunities for targeted clinical intervention. Integrating these findings into routine care pathways could contribute to improved postoperative outcomes in patients with lower and upper leg fractures. In this study, smoking did not demonstrate a strong correlation with complication rates. Nonetheless, targeted screening and the provision of coping strategies to support smoking cessation can still be meaningfully implemented. In our clinical practice, one measure that has already been successfully implemented is the early mobilization of patients. Standard operation procedures were incorporated into postoperative protocols, particularly for geriatric patients. Prompt referral to physiotherapy, individualized mobilization plans, and fall-prevention strategies may help break the cycle of immobilization, sarcopenia, and delayed recovery. A social services team is responsible for coordinating post-discharge care, ensuring optimal linkage to outpatient services and arranging the best possible support in the home environment. The findings of this study have prompted us to initiate a follow-up study focusing on malnutrition as a target for intervention. Early dietetic consultation and protein supplementation offer low-risk, high-benefit interventions that may positively influence complication rates. Given that malnutrition appears to be the most immediately addressable factor, prioritizing nutritional optimization may yield substantial clinical benefit.

Together, these measures support a more holistic approach to fracture management that considers both biological and functional determinants of recovery. Implementing structured pathways may improve patient outcomes, reduce complication rates, and better align clinical practice with future evidence-based risk stratification.

## 5. Limitations

Many of the findings and identified associations are of considerable importance for understanding fracture characteristics and their plausible complications. The broad inclusion criteria result in a representative cohort that reflects everyday hospital practice on standard care wards. A limitation of this study is that, due to the inclusion criteria, patients with only minor injuries not warranting admission, as well as those with severe polytrauma whose primary injuries were located in other body regions, are likely underrepresented. This study ultimately included 416 patients, and the overall sample size was adequate for preliminary association analyses. However, several subgroup analyses were based on comparatively small sample sizes. These limited subgroup sizes may have reduced the statistical power and, consequently, the reliability of certain observed associations—such as the potentially “protective” effect of obesity on complication rates. Therefore, the results of these subgroup analyses should be interpreted with caution. Moreover, the inherent limitations of subgroup analyses, including increased susceptibility to random variation and the risk of over-interpreting findings based on small cohorts, must be acknowledged. Further studies with larger and more balanced subgroups are needed to validate these preliminary observations.

The limited sample size currently prevents meaningful generalization of these findings, and they are not yet suitable for integration into robust predictive modeling frameworks.

## 6. Conclusions

This study does not focus solely on a specific subpopulation but instead captures a broad spectrum of patients. This provides a comprehensive overview of the patient population with surgically managed femoral and lower leg fractures. By examining internal and external key factors and postoperative complication rates, the study aims to better understand adverse postoperative courses in hospitalized patients. A key strength of this study is its inclusion of a broad and representative patient cohort, providing a solid foundation for future investigations, including meta-analyses. But further stratification at a more granular level is not statistically justifiable due to the small sizes of the subgroups; while certain statistical effects remain detectable, these cannot be generalized with confidence. The calculation of individual risk based on established and standardized classification systems could, in the future, be used to allocate available resources more effectively.

Some factors may offer more promising targets for future interventions than others, which could help guide clinical decision-making and optimize resource allocation. Interestingly, obesity demonstrated a negative correlation with complications. Smoking showed only a weak correlation among patients with lower leg fractures and no association in those with femoral fractures. Reduced mobility was significantly associated with an elevated risk of complications. Malnutrition, likewise significantly associated with increased complication rates, may represent the most readily addressable modifiable risk factor and should, therefore, be prioritized for early identification and intervention. From a national health economics perspective, such risk calculations may also help identify high-risk, resource-intensive cases at an early stage, enabling referral to highly specialized centers. Within an in-hospital strategy, institutions could implement measures such as increasing the nurse-to-patient ratio or enhancing consultation frequency for high-risk patients to optimize the allocation of clinical resources. In a forthcoming study, our findings will serve as the basis for developing a predictive model.

## Figures and Tables

**Table 1:** Descriptive data for the total cohort and subgroups of patients with upper and lower leg fractures. The *p*-value indicates whether the differences between the two subgroups are statistically significant.

Characteristics	All	Upper Leg	Lower Leg	*p*-Value
	*N* = 416	*N* = 119	*N* = 297	
Age (years)	54.4 ± 18.3	65.3 ± 19.2	50.1 ± 16.0	0.009
Men	241 (57.9%)	59 (49.6%)	182 (61.3%)	0.012
Height (cm)	172.9 ± 9.6	171.1 ± 10.9	173.7 ± 8.9	0.009
BMI (kg/m^2^)	26.7 ± 4.8	25.3 ± 4.8	27.2 ± 4.8	0.87
Smoking status				
non smoker	212 (51.0%)	68 (57.1%)	144 (48.5%)	0.025
smoker	204 (49.0%)	51 (42.9%)	153 (51.5%)	
Mobility				
unrestricted	326 (78.4%)	75 (63.0%)	251 (84.5%)	<0.001
restricted	90 (21.6%)	44 (37.0%)	46 (15.5%)	
CCI				
0 points	293 (70.4%)	57 (47.9%)	236 (79.5%)	
1–2 points	88 (21.2%)	43 (36.1%)	45 (15.2%)	
3–4 points	22 (5.3%)	13 (10.9%)	9 (3.0%)	<0.001
>4 points	13 (3.1%)	6 (5.0%)	7 (2.4%)	
NRS				
NRS < 3	332 (79.8%)	76 (63.9%)	256 (86.2%)	<0.001
NRS ≥ 3	84 (20.3%)	43 (36.1%)	41 (13.8%)	

**Table 2:** Severity of injury and complication rates in upper and lower leg fractures. The *p*-value indicates whether the differences between the two subgroups are statistically significant.

Characteristics	All	Upper Leg	Lower Leg	*p*-Value
	*N* = 416	*N* = 119	*N* = 297	
Severity of injury according to AIS				
Minor	89 (21.4%)	23 (19.3%)	66 (22.2%)	
Moderate	227 (54.6%)	31 (26.1%)	196 (66.0%)	
Major	100 (24.0%)	65 (54.6%)	35 (11.8%)	<0.001
Serious	0 (0%)	0 (0%)	0 (0%)	
Clavien–Dindo				
Clavien–Dindo 0	202 (48.2%)	49 (41.2%)	153 (51.5%)	
Clavien–Dindo 1	89 (21.4%)	19 (16.0%)	70 (23.6%)	
Clavien–Dindo 2	57 (13.7%)	25 (21.0%)	32 (10.8%)	
Clavien–Dindo 3	43 (10.3%)	8 (6.7%)	35 (11.84%)	<0.001
Clavien–Dindo 4	25 (6.0%)	18 (15.1%)	7 (5.9%)	
Clavien–Dindo 5	0 (0.0%)	0 (0.0%)	0 (0.0%)	
Complications *n* (%)	125 (30.0)	51 (42.9)	74 (24.9)	<0.001

**Table 3:** Correlation analysis of common comorbidities and severity of injury.

Characteristics	All		Upper Leg		Lower Leg	
	Correlation Coefficient	*p*-Value	Correlation Coefficient	*p*-Value	Correlation Coefficient	*p*-Value
Hypertension	0.03	0.50	0.19	0.040	−0.08	0.19
CHD/AP	−0.01	0.93	0.01	0.96	−0.09	0.12
Heart failure	0.02	0.64	0.14	0.13	−0.09	0.12
Cardiac arrhythmia	0.01	0.95	0.07	0.42	−0.07	0.21
PAD	0.02	0.69	0.07	0.43	−0.01	0.99
CVI	0.02	0.64	0.05	0.57	−0.04	0.50
COPD	−0.04	0.44	0.06	0.53	−0.12	0.032
Asthma	−0.04	0.41	−0.09	0.31	−0.02	0.69
Gastritis	0.01	0.85	0.06	0.53	−0.03	0.63
Chronic inflammatory bowel diseases	−0.09	0.08	0.10	0.29	−0.14	0.016
Pancreatic disease	0.01	0.90	0.10	0.29	−0.02	0.69
Hepatitis	−0.05	0.27	−0.05	0.63	−0.11	0.05
Liver cirrhosis	−0.04	0.43	0.01	0.95	−0.06	0.27
Renal insufficiency	0.03	0.53	0.13	0.15	−0.13	0.023
Rheumatoid arthritis	0.07	0.19	0.22	0.014	−0.07	0.23
Obesity	−0.13	0.011	−0.28	0.002	−0.06	0.33
Gout	−0.10	0.034	−0.12	0.20	−0.11	0.06
Diabetes mellitus	−0.10	0.042	0.10	0.30	−0.25	<0.001
Hypothyreosis	0.06	0.25	0.19	0.038	−0.07	0.21

Correlation effect size (r): Interpretation (Cohen) 0.10 = small; 0.30 = medium; 0.50 = large.

**Table 4:** Correlation coefficient of patient characteristics, fracture location and severity of injury.

Characteristics	All		Upper Leg		Lower Leg	
	Correlation Coefficient	*p*-Value	Correlation Coefficient	*p*-Value	Correlation Coefficient	*p*-Value
Age	0.10	0.05	0.35	<0.001	−0.16	0.006
Male	0.16	0.001	0.25	0.007	0.08	0.17
Height	−0.14	0.003	−0.31	<0.001	−0.04	0.94
BMI	−0.23	<0.001	−0.30	<0.001	−0.18	0.002
Smoking	−0.06	0.25	−0.02	0.85	−0.07	0.25
Immobility	0.02	0.63	0.15	0.10	−0.14	0.017
CCI	0.02	0.64	0.24	0.008	−0.22	<0.001
Malnutrition	0.16	<0.001	0.17	0.07	0.09	0.14

Correlation effect size (r): Interpretation (Cohen) 0.10 = small; 0.30 = medium; 0.50 = large.

**Table 5:** Odds ratio of patient characteristics, fracture location, and complications.

Characteristics	All		Upper Leg		Lower Leg	
	OR (95% CI)	*p*-Value	OR (95% CI)	*p*-Value	OR (95% CI)	*p*-Value
Age *	0.15	<0.001	0.20	0.030	0.04	0.53
Gender (men)	1.3 (0.8; 1.9)	0.34	1.8 (0.9; 3.7)	0.11	0.9 (0.5; 1.5)	0.65
Height *	−0.09	0.06	−0.15	0.21	−0.05	0.41
BMI *	−0.05	0.27	−0.16	0.08	0.06	0.35
Smoking	1.4 (1.0; 2.3)	0.06	1.2 (0.6; 2.4)	0.67	1.9 (1.1; 3.3)	0.018
Immobility	2.7 (1.7; 4.4)	<0.001	2.9 (1.3; 6.2)	0.007	2.0 (1.0; 3.9)	0.043
CCI	1.6 (1.2; 2.1)	<0.001	1.8 (1.1; 2.8)	0.013	1.2 (0.8; 1.8)	0.29
Malnutrition	2.6 (1.6; 4.3)	<0.001	2.3 (1.1; 4.9)	0.033	2.2 (1.1; 4.4)	0.027

Correlation effect size (r): Interpretation (Cohen) 0.10 = small; 0.30 = medium; 0.50 = large. * Correlation coefficient calculated.

**Table 6:** Differential comorbidity burden between upper and lower leg fracture patients. The *p*-value indicates whether the differences between the two subgroups are statistically significant.

Comorbidities	All	Upper Leg	Lower Leg	*p*-Value
	*N* = 416	*N* = 119	*N* = 297	
Hypertension	138 (33.2)	59 (49.6)	79 (28.3)	<0.001
CHD/AP	39 (9.4)	24 (20.2)	15 (5.4)	<0.001
Heart failure	26 (6.3)	16 (13.4)	10 (3.6)	<0.001
Heart valve disease	52 (4.1)	10 (8.4)	9 (3.2)	0.018
Cardiac arrhythmia	36 (8.7)	21 (17.6)	15 (5.4)	<0.001
Peripheral arterial disease	15 (3.6)	6 (5.0)	9 (3.2)	0.32
CVI	9 (2.2)	6 (5.1)	3 (1.0)	0.019
COPD	8 (1.9)	4 (3.4)	4 (1.3)	0.18
Asthma	16 (3.8)	7 (5.9)	9 (3.0)	0.17
Gastritis	10 (2.4)	4 (3.4)	6 (2.2)	0.42
Chronic inflammatory bowel diseases	7 (1.7)	1 (0.8)	6 (2.2)	0.40
Pancreatic disease	5 (1.2)	1 (0.8)	4 (1.3)	0.67
Hepatitis	4 (1.0)	3 (2.5)	1 (0.3)	0.039
Liver cirrhosis	3 (0.7)	1 (0.8)	2 (0.7)	0.86
Renal insufficiency	17 (4.1)	12 (10.1)	5 (1.7)	<0.001
Rheumatoid arthritis	22 (5.3)	11 (9.2)	11 (3.7)	0.023
Obesity	50 (12.0)	13 (10.9)	37 (12.5)	0.67
Gout	19 (4.6)	8 (6.7)	11 (3.7)	0.18
Diabetes mellitus	42 (10.1)	19 (16.0)	23 (7.7)	0.018
Hypothyreosis	62 (14.9)	31 (26.1)	31 (10.4)	<0.001

**Table 7:** Odds ratio for common comorbidities in relation to complications.

Characteristics	All		Upper Leg		Lower Leg	
	OR (95% CI)	*p*-Value	OR (95% CI)	*p*-Value	OR (95% CI)	*p*-Value
Hypertension	1.6 (1.0; 2.5)	0.040	1.4 (0.7; 3.0)	0.34	1.4 (0.8; 2.5)	0.22
CHD/AP	3.9 (1.8; 8.4)	<0.001	2.6 (1.0; 6.8)	0.06	4.0 (1.0; 15.2)	0.044
Heart failure	7.2 (2.5; 20.5)	<0.001	6.1 (1.2; 30.3)	0.026	6.5 (1.6, 26.6)	0.010
Cardiac arrhythmia	2.3 (1.1; 5.0)	0.030	1.9 (0.6; 5.9)	0.26	2.1 (0.7; 6.1)	0.17
CVI	1.2 (0.3; 4.7)	0.83	0.7 (0.1; 3.7)	0.63	1.5 (0.1; 16.9)	0.74
COPD	1.4 (0.3; 6.0)	0.64	1.3 (0.2; 10.0)	0.77	1.0 (0.1; 9.8)	1.00
Asthma	1.1 (0.4; 3.1)	0.92	1.8 (0.4; 8.6)	0.44	3.7 (0.1; 3.0)	0.35
Gastritis	-	0.16	4.2 (0.4; 41.5)	0.22	1.5 (0.3; 8.4)	0.63
Chronic inflammatory bowel diseases	0.9 (0.2; 4.9)	0.93	-	-	0.6 (0.1; 5.2)	0.64
Pancreatic disease	1.6 (0.3; 9.5)	0.63	0.0 (0.0; 0.0)	1.00	3.1 (0.4; 22.2)	0.27
Hepatitis	7.1 (0.7; 69.2)	0.09	2.7 (0.2; 31.0)	0.41	-	0.08
Liver cirrhosis	4.7 (0.4; 52.5)	0.21	-	-	3.0 (0.2; 49.2)	0.43
Renal insufficiency	4.6 (1.7; 12.7)	0.003	4.6 (1.1; 18.2)	0.027	2.0 (0.3; 12.4)	0.44
Rheumatoid arthritis	1.4 (0.6; 3.3)	0.50	1.1 (0.3; 3.9)	0.86	1.1 (0.3; 4.5)	0.84
Obesity	0.3 (0.1; 0.7)	0.005	−0.30	<0.001	0.6 (0.2; 1; 4)	0.20
Gout	1.7 (0.7; 4.1)	0.25	7.9 (1.8; 3.5)	0.75	2.6 (0.8; 8.9)	0.12
Diabetes mellitus	2.1 (1.1; 4.0)	0.026	2.1 (0.8; 6.5)	0.15	1.7 (0.7; 4.1)	0.26
Hypothyreosis	1.7 (1.0; 3.0)	0.06	1.9 (0.8; 4.4)	0.12	1.1 (0.5; 2.5)	0.90

## Data Availability

The datasets used and/or analyzed during the current study are not publicly available due to legal restrictions, privacy considerations, and ethical concerns, as they contain sensitive participant information. However, the data are available upon reasonable request and with appropriate permission, subject to a data use agreement, and can be requested from the corresponding author.

## Abbreviations

The following abbreviations are used in this manuscript:

AIS	Abbreviated Injury Scale
AO/OTA	AO Foundation and the Orthopedic Trauma Association
CCI	Charlson Comorbidity Index
CDC	Clavien-Dindo Classification
NRS	Nutritional Risk Screening 2002
